# Legal risks in medical practice

**Published:** 2019-09-10

**Authors:** Vincent K Mutai

**Affiliations:** 1 Advocate of the High Court of Kenya, Nairobi, Kenya.

**Did you know that informed consent can be a defence in claims of negligence? An under-standing of the law is vital in medical practice.**

**Figure:**
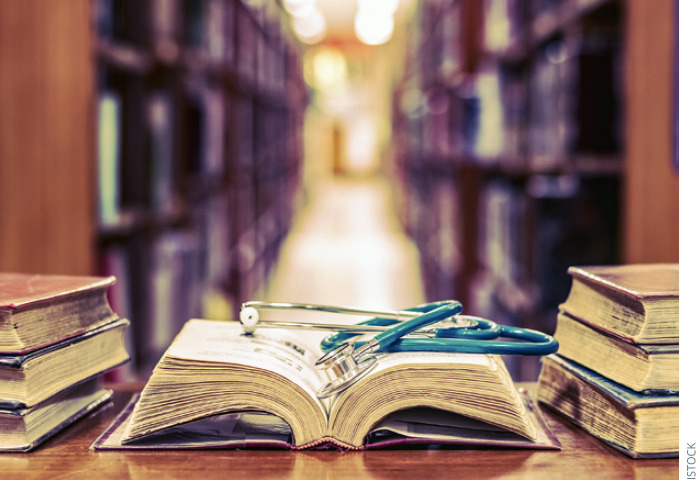
Understanding the law in your country can be reassuring.

Laws are different in different countries, so it is impossible to write a definitive medico-legal guide that will apply in every country. This article sets out some simplified explanations of important medico-legal concepts that are widely accepted around the world.

## Competence and regulation

Patients attend a medical practice believing that the health workers possess skill and expertise that can cure, or at least improve, their medical condition. The patient believes that health workers have a level of skill and competence beyond that of an ordinary person in the street. These skills and competences are acquired through training and experience.

In most countries, doctors and nurses are registered with national regulatory authorities, which confirms that the health worker has sufficient skill and knowledge to treat patients competently.

Regulatory authorities can remove health workers from the register if they are thought to be incompetent and a danger to the public. Alternatively, they may recommend further training, or closer supervision, until the health worker has demonstrated that they are once again competent to deliver care.

## Negligence

Medical treatment carries a risk of causing harm that may lead to the patient being even worse than they were before they were treated. Even if no treatment is provided (perhaps because the condition was not recognised) this may also be harmful. If a patient comes to harm as a result of a health worker's action, or inaction, this may lead to a claim for negligence.

**“Medical treatment carries a risk of causing harm that may lead to the patient being even worse than they were before treatment.”**

Negligence is a claim under private law that arises when a person suffers damages or injury as a result of a failure by another person to exercise care.

To succeed in a negligence claim, the patient must establish that the doctor (or other health worker) failed to take the care that a reasonable doctor would exercise in the same circumstances; this is known as the ‘reasonable person in similar circumstances’ test.

All medical interventions carry a risk that the patient's condition may be worse, rather than better, after treatment. This does not mean that the health worker has been negligent, provided that he or she has exercised reasonable care.

## Vicarious liability

Vicarious liability means liability arising from the action of others under the control or supervision of a senior health worker. For the supervising person to be liable, there must be an employment relationship that places one in authority over the other (e.g., head nurse/matron and nurse).

If a health worker under the supervision of a senior medical professional causes harm through a medical error, the supervising doctor or nurse may be held responsible. Health workers should ensure that the practitioners they supervise have the knowledge, skills, and resources to perform their roles competently and safely.

## Informed consent

Kenya's Health Act No. 21 of 2017 requires that patients must give informed consent before they can be treated. A health care provider is required to provide sufficient information to a patient to enable them to participate actively in decisions about their care. The information should be provided in a language that the user under-stands, and takes into account the user's level of literacy.

The UK Supreme Court recently ruled that a doctor's failure to disclose a risk associated with childbirth was negligence. The Court stated that the doctor has a duty to ensure that patients are properly informed about their condition, the range of treatment options available, and the associated risks, before treatment is given.

The legal significance of informed consent is that it can be a defence to claims of negligence. If a patient agrees to a treatment, knowing that it carries a risk of a complication, they cannot claim negligence if they suffer that complication, provided that the health worker has taken reasonable care to avoid the complication.

